# Alice Hamilton: A Legacy of Advancing Occupational Health and Safety Standards

**DOI:** 10.7759/cureus.70218

**Published:** 2024-09-25

**Authors:** Pratik P Tawde, Sonali G Choudhari, Abhay Gaidhane

**Affiliations:** 1 Preventive and Community Medicine, Jawaharlal Nehru Medical College, Datta Meghe Institute of Higher Education and Research, Wardha, IND

**Keywords:** historical vignette, occupational diseases, occupational health, occupational medicine, occupational safety health act

## Abstract

Alice Hamilton was a physician, research scientist, and author from America. She was a prominent figure in the occupational health industry, established the groundwork for safety measures, and was a trailblazer in industrial toxicology. Hamilton received medical training at the University of Michigan Medical School. While living at Hull House in Chicago from 1887 to 1919, she interacted with a wide range of working-class families and learned about the risks they encountered in their daily lives. In 1897, she was appointed as a pathology professor at the Woman's Medical School of Northwestern University. In 1919, she made history as the initial female faculty member of Harvard University. Furthermore, Hamilton researched mercury, carbon monoxide, rubber, and the munitions industries, in addition to her authoritative work on hazardous lead trades, such as smelting, refining, painting, and manufacturing. She wrote more than 80 scientific reports in 40 years. The U.S. Occupational Safety and Health Act was passed three months after she died in 1970.

## Introduction and background

**Figure 1:**
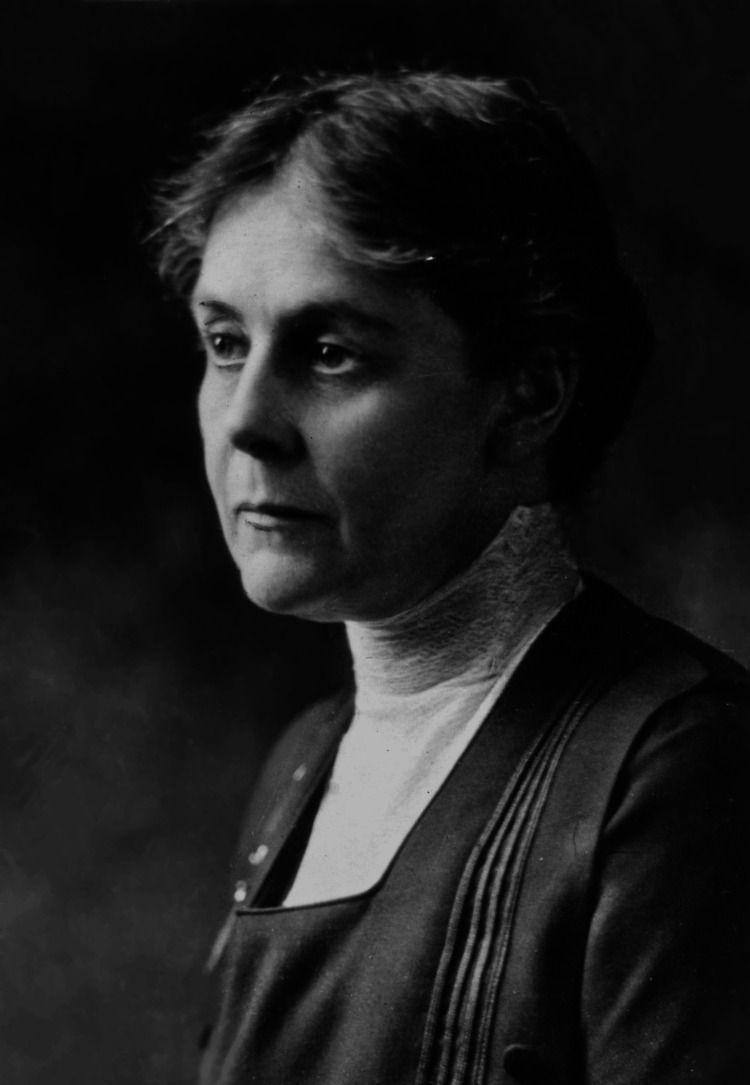
Alice Hamilton Source: [3] Copyright/license: The non-commercial reuse of this content is free and open to all

Alice Hamilton (Figure 1) was an American physician, research scientist, and author who significantly contributed to occupational health. She established the groundwork for safety protocols and was a pioneering figure in the field of industrial toxicology. She was born in 1869 into a well-known family. She completed her medical studies at the University of Michigan in 1893. She began working at the Women's Medical School of Northwestern University in 1897 and also relocated to Hull House. Hamilton ran a clinic for young children in the local community. She started researching occupational illnesses (also known as industrial medicine). Hamilton's pioneering investigations included studies on carbon monoxide poisoning, mercury poisoning, and hand conditions in workers. She later became the first female faculty member at Harvard Medical School, retiring at the age of 66 and continuing her work as a consultant until she died at the age of 101 in 1970 [1,2].

## Review

Early life and education

Alice Hamilton was born in Manhattan in 1869 and grew up in Indiana. Alice's parents taught their five children at home from a young age and later completed her schooling at Miss Porter’s School in 1888 [4]. Before enrolling in medical school, she had a special interest in science and later in anatomy, which she studied at Fort Wayne Medical College [5]. She completed her MD in 1893 from the University of Michigan Medical School. After graduation, she interned for two months at the Women and Children's Hospital in Minnesota and for 10 months at the New England Hospital in Boston [6]. Hamilton went to Germany for one year to become an expert in bacteriology and pathology, as advised by her seniors. Her life was tough in Germany, as universities at that time did not enroll any female students. She also had to face sexism while attending classes and practicals. Hamilton felt bitter about her time in Germany. Alice went back to the U.S. in September 1896 and continued her graduate studies at Johns Hopkins University with Simon Flexner, a young pathologist [7].

Early career and interest in occupational health

In 1897, Hamilton began her career by becoming a faculty member in the Pathology Department at the Women's Medical College of Northwestern University. During these years, she lived at Hull House in Chicago. Hull House was founded by social activists Jane Addams and Ellen Gates Starr. Since she lived among the oppressed working class, experiencing their hardships with injustice, poverty, and illness, she cultivated a passion for social causes and women’s rights. While living at Hull House, she witnessed the negative effects that harsh and unhealthy working conditions had on the men and women she interacted with every day. She also observed disorganized workers dealing with serious issues like low pay, excessive working hours, poverty, and the risk of industrial illnesses, especially from exposure to carbon monoxide and lead. She familiarized herself with industrial diseases using whatever information was available about them [8].

Medical investigator

In 1910, Governor Deneen of Illinois established an Occupational Disease Commission to investigate the prevalence of industrial illness in the state, marking the first survey of its kind in the United States. Dr. Hamilton's extensive professional and social background proved valuable in her role as a member of the commission. The commission focused on a few industrial poisons: lead (Pb), arsenic (As), brass (an alloy of copper and zinc), carbon monoxide (CO), cyanides (CN⁻), and turpentines. Dr. Hamilton wrote the “Illinois Survey,” and the commission’s efforts led to the enhancement of the first workers' compensation laws in Illinois in 1911, Indiana in 1915, and occupational disease laws in other states. In 1916, Hamilton became the United States' foremost expert on lead poisoning [9,10]. She assessed various occupational toxic diseases caused by substances such as mercury, benzene, and poisonous gases. Hamilton was employed by different state and federal health committees for the next 10 years. In 1925, during a conference of the Public Health Service, she spoke out against the use of lead in gasoline, raising concerns about its negative impacts on both people and the environment. Her assertions went unrecognized, and it was estimated in 1988 that more than 68 million children were exposed to lead from the fuel [11]. Following this, Dr. Alice began investigating and working for the U.S. Bureau of Labor Statistics, focusing on white lead and lead oxide investigations. After gathering information on confirmed poisoning cases and studying them, she helped shape the development of modern toxicology and laboratory practices. Her findings played a crucial role in pushing for health reforms that ultimately led to the establishment of the Occupational Safety and Health Administration [12,13]. Throughout World War I, Dr. Alice aided the U.S. Army by revealing a puzzling sickness that affected laborers in New Jersey. She headed a team that determined workers were facing exposure to trinitrotoluene (TNT), and the issue was resolved by washing clothing at the end of each shift [14]. Hamilton's most famous research involved her investigations into carbon monoxide poisoning among steelworkers in America, mercury poisoning in hatters, and a hand condition affecting workers who used jackhammers [1]. She was asked by the U.S. Department of Labor to look into industries that were producing high explosives and the "dead fingers" condition among limestone cutters.

Assistant professor

In 1919, her recognition as a specialist in industrial medicine resulted in her being named a faculty member at Harvard Medical School. At a time when Harvard Medical School still did not allow women students, she made history as the first female faculty member. Throughout her 16 years at Harvard University, she never once achieved a promotion in faculty rank; she was only given a series of three-year commitments. She was unable to participate in social events and the university's graduation processions. From 1924 to 1930, she was the only woman serving on the League of Nations Health Committee. She visited Hull House annually until Jane Addams passed away in 1935. Following her retirement in 1935, Hamilton kept her job as a medical consultant for the U.S. Division of Labor Standards and stayed connected to Harvard as a professor emerita. A commemorative stamp from the U.S. Postal Service honored her significant contributions to public health in 1995 [1,2].

Dr. Alice Hamilton and her Federal Bureau of Investigation (FBI) file

Dr. Hamilton caught the FBI's attention in 1942 by advocating for clemency for Morris Schappes, a former professor convicted of perjury. By 1950, the FBI had identified her involvement in various communist organizations and causes, such as the Joint Anti-Fascist Refugee Committee and the American Peace Crusade. Despite being monitored for her associations, she refused to implicate others and called for the repeal of laws allowing imprisonment without due process. In 1952, she protested against alleged plans to detain thousands of Communists in concentration camps. Alice Hamilton's defiance of FBI surveillance and support for communist fronts led to ongoing scrutiny, though the FBI never found evidence linking her to the Communist Party. She remained unapologetic for her beliefs and actions, demonstrating unwavering courage in the face of government oppression and surveillance [15].

Mentoring relationship with Harriet Hardy

Alice Hamilton and Harriet Hardy were two women physician-researchers who greatly influenced the advancement of occupational health in the U.S. in the 20th century. Hamilton and Hardy shared many similarities as doctors who were both deeply passionate about the well-being of laborers. Hamilton's interest in occupational health stemmed from her worry about the health issues faced by the poor working class. Hardy, who graduated with her medical degree in Manhattan in 1932, also witnessed the challenges faced by workers and the occupational injuries and illnesses of that era. Hardy and Hamilton started working together in 1946 when Hardy was 40 years old and Hamilton was 77. Hardy worked at both the Massachusetts General Hospital and the Massachusetts Department of Labor and Industries. The publication in 1946 of a case series involving 17 workers in a fluorescent lamp factory who developed "chemical pneumonitis" after being exposed to beryllium was significant, as it was one of the first in the U.S. to link beryllium exposure to lung disease in the industry [16]. Alice Hamilton read Hardy's publication, extended her congratulations, and then invited her to collaborate on the second edition of *Industrial Toxicology*. The two women initially connected through their collaboration in *Industrial Toxicology* and continued to flourish over the years. They exchanged letters and visited each other often. Hamilton consistently provided professional support and career advice to Hardy, praising her accomplishments and encouraging her to excel in academia. Hamilton celebrated Hardy's successes, such as her appointments at the Massachusetts Institute of Technology and Harvard Medical School, and applauded her publications. Hardy sought advice from Hamilton on various career-related matters, highlighting Hamilton's importance in her professional development. Although Hamilton sometimes criticized Hardy, she also encouraged her to take advantage of opportunities and make the most out of life. Their relationship had a strong maternal tone at times, with Hamilton reminding Hardy to prioritize her health and avoid overworking. Overall, Hamilton played a crucial role in guiding Hardy's career and encouraging her to take on new challenges [17].

Death

Hamilton passed away from a stroke at her residence in Connecticut in 1970. The Occupational Safety and Health Act was approved to enhance workplace safety three months after her death [13].

## Conclusions

Hamilton's keen awareness of her circumstances and those of others also encompassed her understanding of the broader relationships between work-related illnesses, lack of financial resources, being an immigrant, and facing social prejudice. This excerpt from her memoir demonstrates her ability to vividly comprehend both the analytical and empathetic aspects of how immigrant workers in America were often taken advantage of by their employers due to their hopes for a better life. Hamilton was a dedicated researcher and supporter who opposed the use of harmful and toxic chemicals in work environments. She remained committed to advocating for social reform in civil liberties, peace, birth control, and protective labor laws for women until her death.

## References

[1] (2024). Alice Hamilton. 202420161230141742.

[2] (2024). Changing the face of medicine: Dr. Alice Hamilton. https://cfmedicine.nlm.nih.gov/physicians/biography_137.html.

[3] Institution S: Alice Hamilton (2024). Alice Hamilton (1869-1970). https://www.flickr.com/photos/smithsonian/5494401632/.

[4] Sicherman B (2013). A Life in Letters.

[5] Sicherman B, Green CH (1980). Notable American Women the Modern Period: A Biographical Dictionary. https://archive.org/details/notableamericanw00sich.

[6] Gugin LC, Clair JES (2015). Indiana’s 200: The People Who Shaped the Hoosier State. Indiana Historical Society Press.

[7] Sicherman B (1984). Exploring the dangerous trades (1908-1914). Alice Hamilton: A Life in Letters.

[8] Corn JK (1988). Alice Hamilton: an historian’s perspective. J Occup Environ.

[9] Ginsberg J (2024). Alice Hamilton and the Development of Occupational Medicine. https://www.acs.org/content/dam/acsorg/education/whatischemistry/landmarks/alicehamilton/alice-hamilton-and-the-development-of-occupational-medicine-commemorative-booklet.pdf.

[10] (2024). A voice in the wilderness: Alice Hamilton and the Illinois survey. https://blogs.cdc.gov/niosh-science-blog/2014/04/28/alice-hamilton/.

[11] Kovarik W (2005). Ethyl-leaded gasoline: how a classic occupational disease became an international public health disaster. Int J Occup Environ Health.

[12] Harbison RD, Bourgeois MM, Johnson GT (2015). Hamilton and Hardy's Industrial Toxicology. Hamilton and Hardy’s Industrial Toxicology. John Wiley & Sons.

[13] Luma M (2021). Women in toxicology in the United States. Toxicol Res (Camb).

[14] Baron SL, Brown TM (2009). Alice Hamilton (1869-1970): mother of US occupational medicine. Am J Public Health.

[15] Castleman B (2016). Alice Hamilton and the FBI. Int J Occup Environ Health.

[16] Hardy HL, Tabershaw IR (1946). Delayed chemical pneumonitis occurring in workers exposed to beryllium compounds. J Ind Hyg Toxicol.

[17] Sullivan M (2017). Hamilton and Hardy: mentoring and friendship in the service of occupational health. Public Health Rep.

